# Cannabis Indica in Hemicrania

**Published:** 1881-02

**Authors:** 


### CANNABIS INDICA IN HEMICRANIA.

FROM an extended experience with *cannabis indica in hemicrania*, the author concludes that the drug is useful in the variety of the affection which is attended with arterial spasm—the same class of cases in which nitrite of amyle is beneficial. The curative effect of hemp in these cases is due to its action as a vascular dilator. In the opposite class of cases—the "neuro-paralytic variety of hemicrania"—attended with flushed face and throbbing temples, cannabis indica does harm: ergot is more applicable to this latter class. The diagnosis made, the extract of cannabis indica should be given for six months at least, one-quarter to three-quarters of a grain three times a day. [The possibility of establishing a habit by so continued an administration is not mentioned by the author."]

